# Supplementary material for: Dietary restriction of amino acids for Cancer therapy
Source: Nutr Metab (Lond). 2020 Mar 14;17:20. doi: 10.1186/s12986-020-00439-x (PMC7071719; doi:10.1186/s12986-020-00439-x)
Supplement: Supplementary file 4 — Additional file 4. [file 12986_2020_439_MOESM4_ESM.pdf]

		Median	SD	3-sigma limit	ERP* Number
**Alanine**	**A**	6.71	3.26	16.49	969
**Cysteine**	**C**	1.93	2.30	8.83	1195
**Asparate**	**D**	4.54	2.29	11.42	754
**Glutamate**	**E**	6.45	3.44	16.78	918
**Phenylalanine**	**F**	3.49	2.15	9.95	836
**Glycine**	**G**	6.32	3.36	16.40	1041
**Histidine**	**H**	2.38	1.65	7.34	1092
**Isoleucine**	**I**	4.06	2.38	11.20	588
**Lysine**	**K**	5.26	3.27	15.08	918
**Leucine**	**L**	9.90	3.71	21.02	604
**Methionine**	**M**	2.27	1.66	7.26	1258
**Asparagine**	**N**	3.29	1.97	9.20	705
**Pronine**	**P**	5.55	3.63	16.44	1293
**Glutamine**	**Q**	4.40	2.49	11.88	918
**Arginine**	**R**	5.55	2.95	14.39	1092
**Serine**	**S**	7.69	3.47	18.11	1108
**Threonine**	**T**	5.05	2.38	12.20	915
**Valine**	**V**	5.88	2.50	13.38	595
**Tryptophan**	**W**	1.17	1.30	5.06	1298
**Tyrosine**	**Y**	2.47	1.78	7.81	756

* ERP (Exceptional rich protein): AA enrichment > 3-sigma upper limit
